# Inequalities in the Oral Health-Related Quality of Life Among Children in Saudi Arabia

**DOI:** 10.7759/cureus.49456

**Published:** 2023-11-26

**Authors:** Omar S Almajed, Haya Alayadi, Wael Sabbah

**Affiliations:** 1 Pediatric Dentistry, Imam Abdulrahman Bin Faisal University, Dammam, SAU; 2 Dental Public Health, King’s College London, London, GBR; 3 Dental Health, College of Applied Medical Sciences, King Saud University, Riyadh, SAU

**Keywords:** untreated caries, inequalities in oral health, dental caries, ohrqol, oral health-related quality of life

## Abstract

Objective: This study aims to examine the Oral Health-Related Quality of Life (OHRQoL) and its determinants among elementary school children in Saudi Arabia, recognizing OHRQoL as a critical aspect of overall health and well-being.

Background: OHRQoL is an essential element of health, influencing children’s ability to engage in daily activities, learning, and social interactions. In Saudi Arabia, despite free dental care, significant occurrences of untreated dental caries among children highlight disparities in oral health outcomes, likely influenced by socioeconomic factors.

Method: Baseline data from a longitudinal randomized controlled trial conducted in Riyadh, Saudi Arabia was utilized. Participants were elementary school students attending public schools, selected using stratified cluster random sampling. The study focused on both deciduous and permanent dentition, excluding children with medical issues. Data collection involved clinical evaluations and parental questionnaires, adhering to WHO criteria.

Results: The results of the study revealed significant associations between age (mean: 98.99 months, 95% confidence interval (CI): 97.8-100.1) and untreated caries (mean: 2.54, 95% CI: 2.34-2.74) with OHRQoL among children in Saudi Arabia. Older children (Rate Ratio (RR) = 1.01; 95% CI: 1.01-1.06) and those with untreated caries (RR = 1.04; 95% CI: 1.01-1.07) had higher rates of experiencing suboptimal oral health outcomes. However, no statistically significant associations were found for other variables such as gender, family income, parental education, oral hygiene frequency, and dental visits with respect to OHRQoL.

Conclusion: The study underscores that age and untreated caries are significantly and positively associated with OHRQoL in children. These findings point to the need for targeted oral health interventions and policies within the sociocultural context of Saudi Arabia, particularly focusing on early prevention and addressing socioeconomic inequalities.

## Introduction

The World Health Organization (WHO) recognizes the Oral Health-Related Quality of Life (OHRQoL) as an essential element of overall health and well-being. The Global Oral Health Programme has been recognized by the WHO as a crucial component [1]. According to the United States Department of Health and Human Services (DHHS), OHRQoL is a complex construct that encompasses various dimensions, including individuals' level of comfort during activities such as eating, sleeping, and socializing, individual's self-esteem and overall satisfaction with their oral health [2]. Furthermore, OHRQoL can profoundly impact children's lives, influencing the ability to engage in day-to-day activities and learning, school performance, and social interactions. Dental caries, malocclusion, and other oral diseases can cause discomfort, eating difficulties, and embarrassment, thus reducing the child's quality of life [3-5]. Therefore, addressing and preventing such problems from an early age is crucial.

Unfortunately, inequalities in OHRQoL often reflect broader societal disparities. It is essential to explore specific factors that can influence children’s oral health outcomes, including inequality and other associated factors. Research has shown that parental education, income, and occupation are closely related to the OHRQoL of the children [6]. Children from economically disadvantaged households are at a higher risk of experiencing suboptimal oral health outcomes. This can be attributed to a multitude of factors, including but not limited to the limited availability of dental care services, bad dietary habits, and insufficient education regarding oral hygiene practices. These inequalities can compound over time, resulting in considerable differences in children's OHRQoL [7]. Gender may also play a role in OHRQoL. Multiple research studies have concluded that male individuals exhibit a superior OHRQoL compared to their female counterparts [8]. The observed phenomenon may be attributed to comparatively lower levels of self-esteem and a more pessimistic outlook toward oral health and body image among females in contrast to males [8].

Saudi Arabia, a wealthy nation providing free dental care services, still struggles with the significant occurrence of untreated dental caries among children [9-11]. According to Ministry of Health data, 96% and 93.7% of Saudi Arabian children between the ages of six and twelve have dental caries [12]. Despite the availability of free dental services, an inequality in their utilization remains evident [13]. Accessibility issues such as long waiting lists, limited available procedures, and perceptions of superior quality care provided by private dental clinics explain the underutilization of dental services in Saudi Arabia.

This research proposes that socioeconomic inequalities profoundly influence the OHRQoL in Saudi Arabian children. It is postulated that children from socioeconomically disadvantaged backgrounds will likely have lower OHRQoL, mainly due to a higher prevalence of dental caries and less frequent use of available dental care services. This hypothesis is supported by existing literature. For instance, Knorst et al., in a systematic review, concluded that individuals of low socioeconomic status (SES) had poorer OHRQoL, regardless of the country’s economic classification [14].

Further justification for this research lies in its potential to inform and influence oral health policies, intervention strategies, and educational efforts within Saudi Arabia. By providing valuable insights into socioeconomic factors impacting children's OHRQoL, this study could contribute significantly towards tailoring effective preventative and intervention approaches to the unique sociocultural context of Saudi Arabia. Furthermore, cultural practices, dietary habits, and societal attitudes towards oral health in the Kingdom could also serve as vital components in the complex web of factors influencing children's OHRQoL.

Baseline data from an intervention study conducted in Riyadh, Saudi Arabia, between 2017 and 2018 were utilized for this research. The study sample was randomly chosen for children aged 6 to 12 years from 16 schools in Riyadh. This investigation will examine OHRQoL among the sample, considering socioeconomic inequalities and other associated factors. By examining both inequality and other relevant factors, this research aims to address the information gap regarding the OHRQoL of children in Saudi Arabia.

## Materials and methods

Ethical approval

The research was granted approval by the Research Ethics Subcommittee for Biomedical Science, Dentistry Medicine, and Natural and Mathematical Science at King's College London, with reference number HR-16/17-4683. Moreover, the study obtained further approval from King Abdelaziz City for Science and Technology (H-01R-012) and the Ministry of Education. Additionally, written informed consent forms were signed by both parents and participants.

Study population

The author of the study utilized baseline data from a longitudinal randomized controlled trial conducted over a period of four months in Riyadh, Saudi Arabia [15]. The elementary school students who were enrolled in public schools constituted the study participants. The study utilized a stratified cluster random sampling approach to randomly choose 16 schools from a list supplied by the Ministry of Education. The researchers recruited participants within the age range of six to twelve years. The age group selection was made with the intention of facilitating a more comprehensive comprehension of the frequency of untreated dental caries in deciduous teeth, while also enabling the observation of the emergence of permanent teeth. The research was centred on the examination of both deciduous and permanent dentition. The study excluded children who had any medical conditions. The study encompassed a sample size of 1086 participants, and data was collected through the utilization of clinical assessments and parental surveys.

The research project utilized the criteria outlined by the WHO to assess the oral health status of the study participants [16]. In addition, the researchers utilized a modified version of the WHO's parental survey to collect data pertaining to the demographic and socioeconomic characteristics (such as age, gender, monthly income, and educational background of both parents) and conduct of the cohort under investigation. The primary outcomes of significance in this investigation are the OHRQoL and the sociodemographic variables. The study assessed the participants' SES through a six-choice question that inquired about their monthly household income. The options ranged from less than 5000 SR to 10,000 SR or over.

Additionally, parents' educational background data was collected as a sociodemographic measure. The educational level of the parents was considered. The survey posed a uniform question to each parent, offering nine distinct options as responses. These options included no formal education, less than primary school education, completion of primary school, attainment of intermediate or middle school education, acquisition of a high school diploma, completion of a college or university degree, possession of postgraduate qualifications, absence of an adult male or female in the household, or lack of awareness/unknown status.

OHRQoL

The key outcome variable in this study is OHRQoL, measured using a series of six questions. These questions are designed to assess various aspects of children's oral health, including their perception of dental aesthetics, the social and functional impacts of oral health, and its influence on behaviour and school attendance. The questions collectively gather information about different elements of oral health and their impact on overall well-being.

Respondents could answer each question with 'yes,' 'no,' or 'do not know.' OHRQoL in this study was quantified as a count variable, where each question was assigned a numerical value (0 for 'no' or 'do not know' and 1 for 'yes'). The total score for each participant, ranging from 0 to 6, represents the overall impact of oral health on their quality of life. This scoring system facilitates a quantitative assessment and allows for detailed statistical analysis.

For further information, the complete questionnaires in both English and Arabic are provided in the appendices of this study.

## Results

The sample included in the analysis consisted of 808 children included in Table 1. The sample consisted of children, with 18.32% identified as male and 81.68% as female. The age of the participants has a mean value of 98.99 months, with a 95% confidence interval (CI) ranging from 97.8 to 100.1.

**Table 1:** Distribution of the variables in the sample included in the analysis among primary school children in Riyadh city in 2017/2018, n=808.

Variable		Percentage/Mean and 95% CI
Gender	Male	18.32%
	Female	81.68%
Age [mean (95% CI)]		98.99 months (97.8-100.1)
Mother Education	Less than high school	22.52%
	High school	33.71%
	College or more	44.31%
Father Education	Less than high school	19.18%
	High school	32.43%
	College or more	48.39%
Family Income Group	Less than 5000	35.27%
	5000-10000	32.92%
	>10000	31.81%
Dental visit within 12 months	Yes	46.66%
	No	53.34%
Oral Hygiene Frequency groups	Never, several times a month, once‎/week	43.32%
	Once‎/day, two or more‎/day	56.68%
Untreated Caries [mean (95% CI)]		2.54 (2.34-2.74)

Regarding the educational background of the children’s mothers, the majority had at least completed high school or more (78.02%), with 22.52% having less than a high school education. Similarly, in the fathers’ education, the majority had at least completed high school or more (80.82%), with 19.18% having less than a high school education.

The sample represents individuals from different family income groups, with the majority falling into the “less than 5000” income bracket (35.27%), followed by the “5000-10000” range (32.92%) and the “>10000” category (31.81%).

In terms of dental visits, 46.66% of the children had visited a dentist within the past 12 months, while 53.54% had not.

Regarding oral hygiene practices, most of the children followed a more frequent oral hygiene routine, brushing at least once a day or multiple times a day (56.68%), compared to 43.32% who reported never or infrequent oral hygiene routines (several times a month or once a week).

Lastly, the average number of untreated caries among the children was 2.53, with a CI ranging from 2.34 to 2.74.

The negative binomial regression analysis results presented in Table 2, shed light on the association between various factors and OHRQoL among children in Saudi Arabia. The analysis aimed to explore the impact of socio-demographic variables, oral hygiene practices, and dental visits on children's OHRQoL.

**Table 2:** Negative binomial regression showing rate ratios for factors associated with QoL among primary school children in Riyadh city in 2017/2018, n=808. QoL: quality of life

Variable	RR	P-value	[95% conf. interval]
Sex	0.85	0.21	0.66-1.09
Age	1.01	0.02	1.01-1.06
Family Income Group			
5000-9000	1.03	0.83	0.82-1.29
10000 or more	0.98	0.87	0.77-1.25
Mother Education			
High school	1.26	0.09	0.96-1.65
College or more	1.13	0.41	0.85-1.50
Father Education			
High school	0.95	0.72	0.72-1.25
College or more	0.99	0.95	0.74-1.32
Oral hygiene frequency			
once/day, two or more/day	1.04	0.69	0.86-1.25
Dental visits within 12 months			
Yes	1.03	0.67	0.86-1.25
Untreated caries	1.04	0.02	1.01-1.07

When examining the influence of Gender on OHRQoL, sex did not show a significant association. This implies that there is no significant difference in OHRQoL between boys and girls in the sample. These findings challenge previous research that suggested males exhibit superior OHRQoL compared to females, indicating that this phenomenon may not apply to the specific population of Saudi Arabian children.

On the other hand, age was found to have a significant positive association with OHRQoL. For each additional month of age, there was a 1.01 times increase in the rate of experiencing suboptimal oral health outcomes (Rate Ratio ‘RR’=1.01,95% CI (1.01-1.06)). This suggests that older children were likelier to have lower OHRQoL than younger children in the sample.

Exploring the role of socioeconomic factors, the analysis considered family income and parental education. The results did not indicate a significant association between family income and OHRQoL. Specifically, for the income groups of 5000-9000 SAR, the p-value was 0.83, and for the income group of 10000 SAR or more, the p-value was 0.87. This indicates that the income groups (5000-9000 SAR and 10000 SAR or more) did not substantially impact OHRQoL. This suggests that within this sample of Saudi Arabian children, family income may not be a significant determinant of OHRQoL.

Regarding parental education, the analysis showed different impacts on OHRQoL based on the education levels of the mother and father. For the mother's education, a marginally significant positive association with OHRQoL was observed in the "High school" category. The Relative Risk (RR) for this category was 0.09, with a 95% CI of 0.96-1.65. This suggests that children whose mothers completed high school education may experience slightly better OHRQoL compared to those with lower levels of maternal education. For mothers with "College or more" education, the RR was 0.41, with a 95% CI of 0.85-1.50. In contrast, the father's education did not show a significant association with OHRQoL. The p-value for the "High school" category was 0.72, and for "College or more," it was 0.95. This indicates that the father's education level may not substantially impact OHRQoL in our sample.

Oral hygiene frequency, measured by the frequency of brushing teeth and dental visits within the past 12 months, also did not show a significant association with OHRQoL.

An essential finding from the regression analysis is the significant positive association between untreated caries and OHRQoL. Children with untreated dental caries were likelier to have lower OHRQoL than those without untreated caries. For each unit increase in the number of untreated caries, the rate of experiencing suboptimal oral health outcomes increased by a factor of 1.04 (RR=1.04, 95% CI (1.01-1.07)). This suggests that children with untreated caries were likelier to have lower OHRQoL than those without untreated caries.

In summary, age and untreated caries were found to be significant factors associated with OHRQoL. Older children and those with untreated caries had higher rates of experiencing suboptimal oral health outcomes. The other variables, including gender, family income, parental education, oral hygiene frequency, and dental visits, did not significantly affect OHRQoL in the sample population.

## Discussion

Age and untreated caries were found to have a positive association with OHRQoL. In the findings, older children and children with untreated dental caries had lower OHRQoL than their younger counterparts and those without untreated dental caries. The rate of children experiencing suboptimal oral health outcomes increases with age and the period children with untreated caries live with that oral condition. These findings are consistent with other research showing that dental caries and greater age are associated with adverse child and family experiences and lower OHRQoL [8].

Several studies have demonstrated that socioeconomic inequalities profoundly influence the OHRQoL in children. For example, Knorst et al. concluded that low SES is associated with worse OHRQoL in all age groups, regardless of their country’s economic classifications, and parental income level and occupation are closely related to a child’s OHRQoL [6,14]. Additionally, recent research conducted among Saudi male teenagers reinforces these findings and highlights the importance of oral health practices, such as fluoride toothpaste, in reducing caries and improving OHRQoL [17]. These collective findings underscore the need for targeted interventions and policies to address socioeconomic inequalities and promote oral health practices for better oral health outcomes among children. Other existing studies have also demonstrated the impact of family, oral hygiene practices, and dental visits on children's OHRQoL. For example, worsening child and family quality of life increases the child’s experience with dental caries and inequalities in the utilization of dental services leading to significant differences in children's OHRQoL [10].

In the present study, the strength of the associations between socioeconomic factors of family income and parental education and OHRQoL slightly varied. The findings demonstrate family income and parental education, among other variables or factors surrounding socioeconomic aspects, are associated with each other. For instance, the results are consistent in that the effect of socioeconomic inequalities in OHRQoL is attributed to mediating factors of oral health behaviours or oral hygiene frequency, self-esteem, parental education, and utilization of oral health care services or dental visits rather than family income [7]. These inconsistencies with prior research could be attributed to various factors. The selection of participants from public schools could have limited socioeconomic diversity, obscuring potential inequalities. Moreover, the use of a simplified OHRQoL questionnaire might have overlooked complex interplays between socioeconomic factors and oral health outcomes.

Limitations

Our study, while providing valuable insights, has certain limitations. Its cross-sectional nature, a common approach in epidemiological studies, limits our ability to establish causality or temporal relationships. Additionally, the gender composition, with a higher proportion of females, might introduce a gender bias, which could affect the generalizability of our findings. Reliance on parent-reported data, while practical in pediatric research, may introduce recall bias. Lastly, the use of the simplified WHO OHRQoL questionnaire could potentially overlook nuanced aspects of oral health specific to Saudi Arabian children.

Future implications

This cross-sectional study highlights areas for further exploration in oral health research. While our approach provides a valuable snapshot of oral health outcomes among Saudi Arabian children, subsequent studies might benefit from employing a longitudinal design to unravel causal relationships and track changes over time. Future research should aim for a more gender-balanced sample to deepen the understanding of gender-specific oral health needs. Additionally, incorporating both child-reported and parent-reported data could offer a more nuanced perspective on children's oral health experiences. The development of culturally specific OHRQoL questionnaires tailored to the Saudi Arabian context could further refine our understanding of region-specific oral health impacts. Overall, the findings underscore the importance of designing targeted interventions and informed policies to enhance oral health outcomes in Saudi Arabia.

## Conclusions

Our study revealed that age and untreated dental caries are significant determinants of OHRQoL among Saudi Arabian elementary school children. Specifically, younger children and those with untreated caries were more likely to experience suboptimal oral health outcomes. Additionally, the education level of mothers, particularly completion of high school, was marginally associated with better children’s OHRQoL. In contrast, factors such as gender, family income, father’s education, oral hygiene frequency, and dental visits did not show a significant impact on OHRQoL. Despite the limitation of potential residual confounding, these findings underscore the importance of early oral health interventions in schools, parental involvement in dental health education, addressing inequalities, and improving access and utilization of dental services for this population.

## Figures and Tables

**Figure 1:**
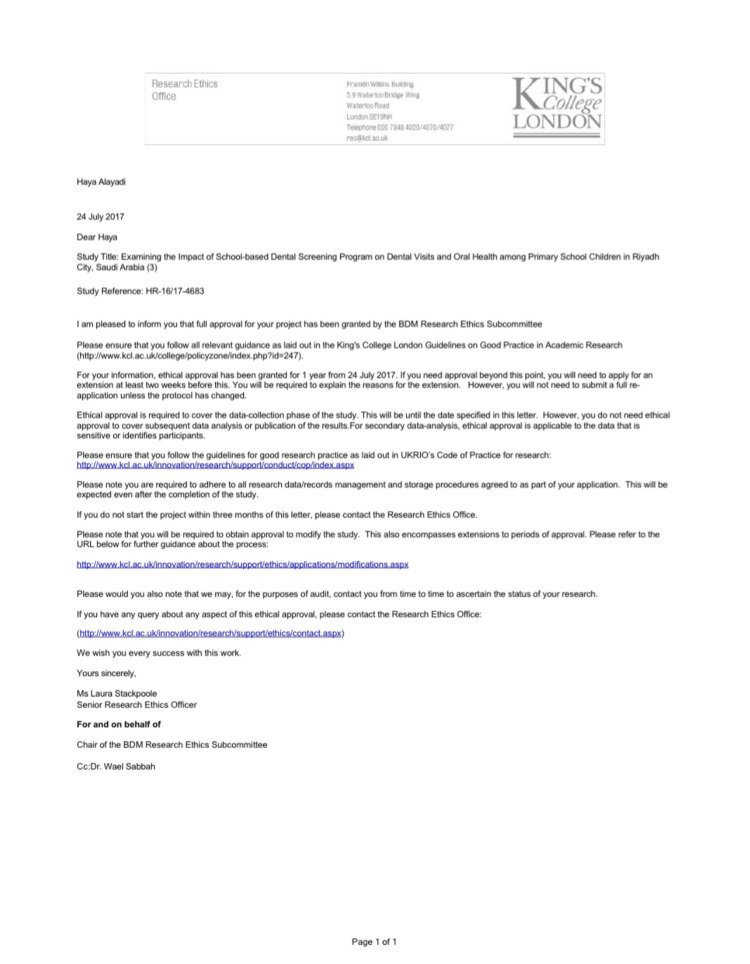
Ethical Approval 1

**Figure 2:**
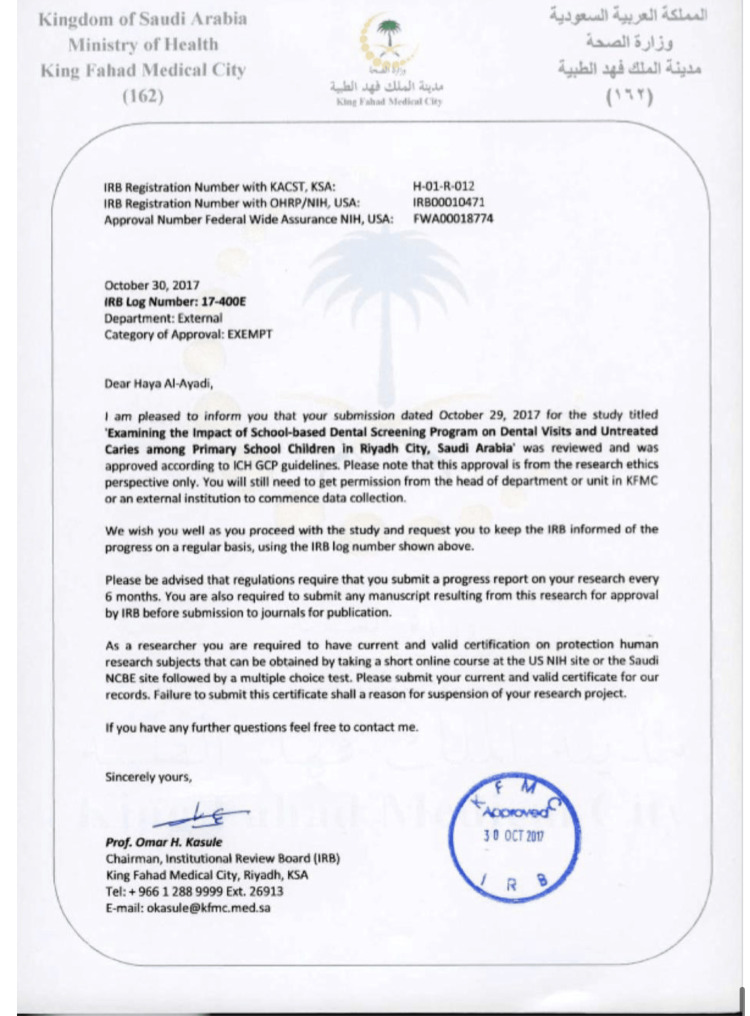
Ethical Approval 2

**Figure 3:**
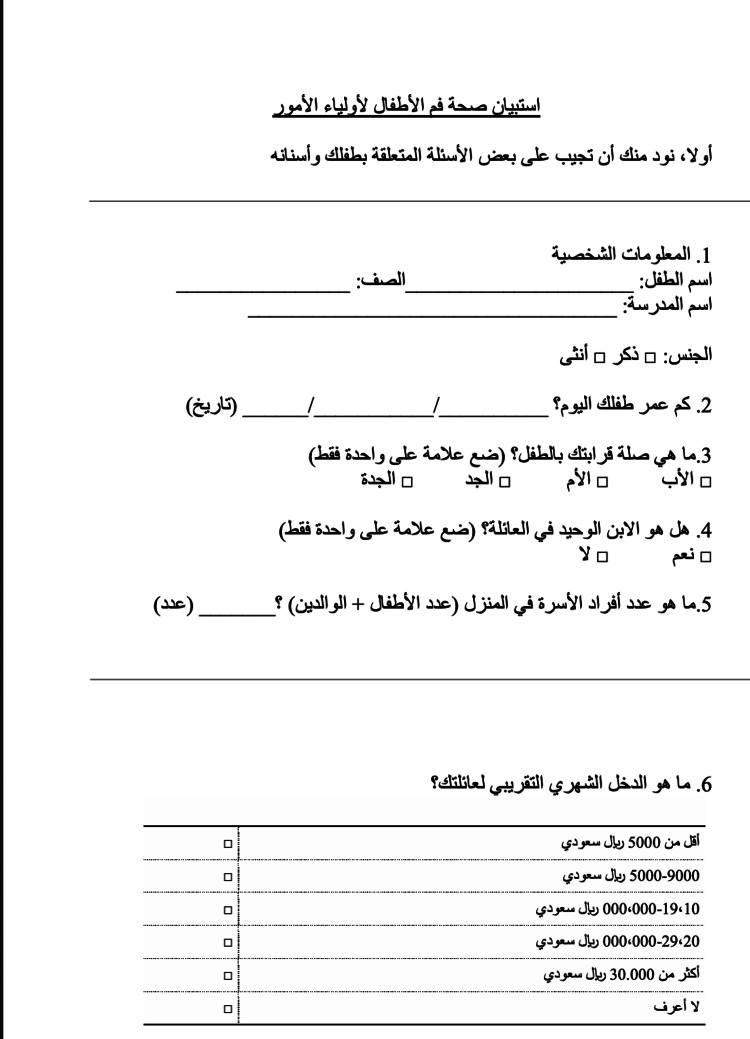
Arabic Questionnaire 1

**Figure 4:**
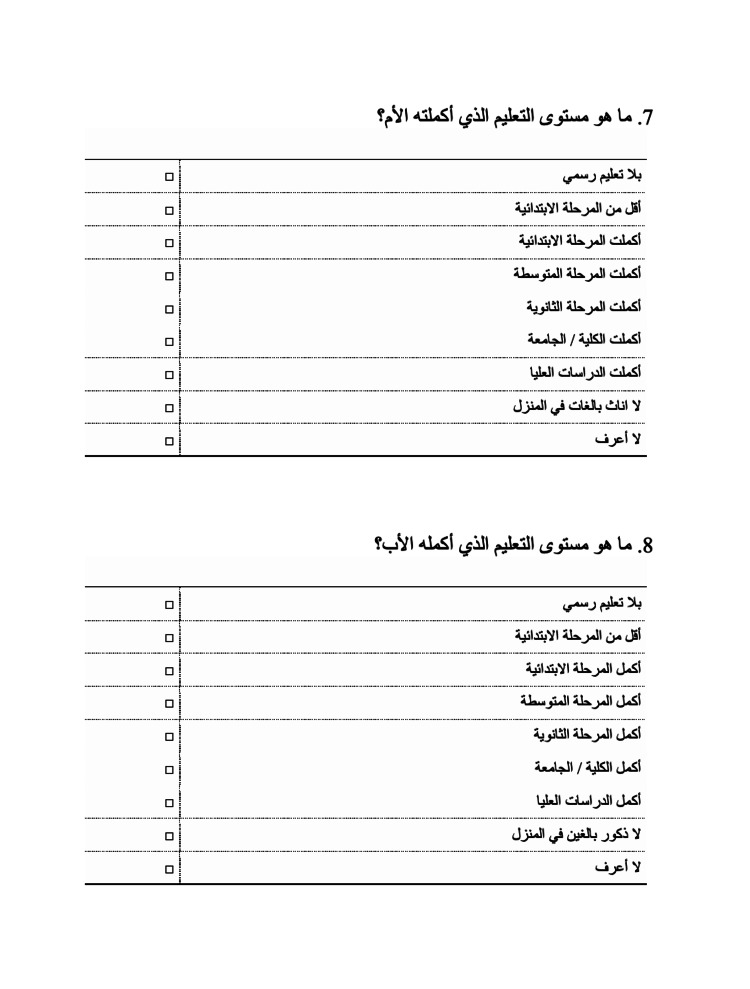
Arabic Questionnaire 2

**Figure 5:**
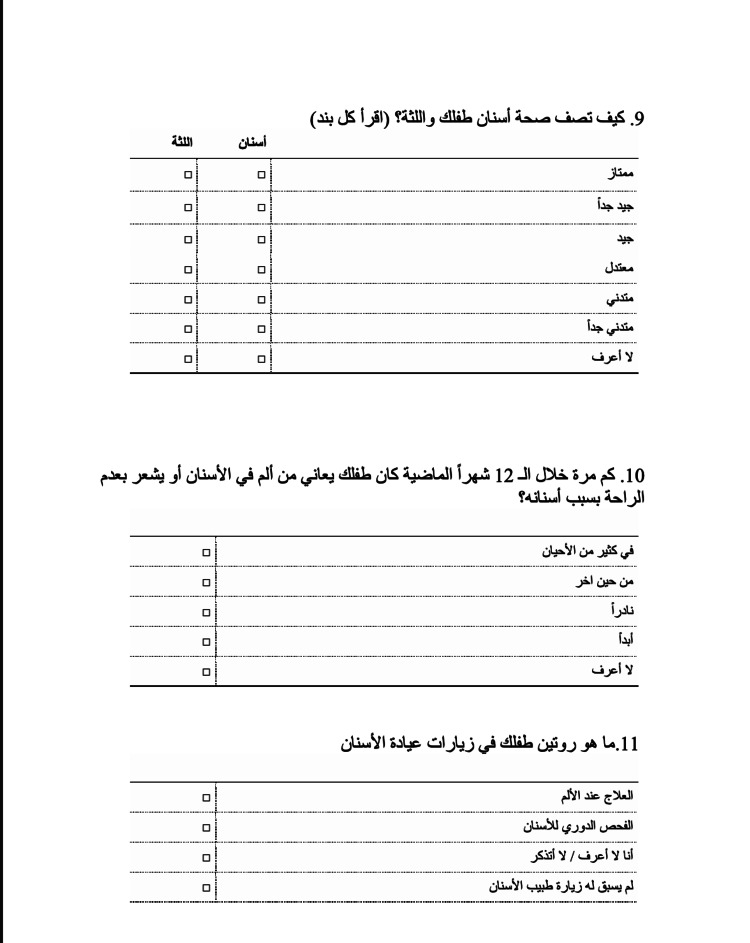
Arabic Questionnaire 3

**Figure 6:**
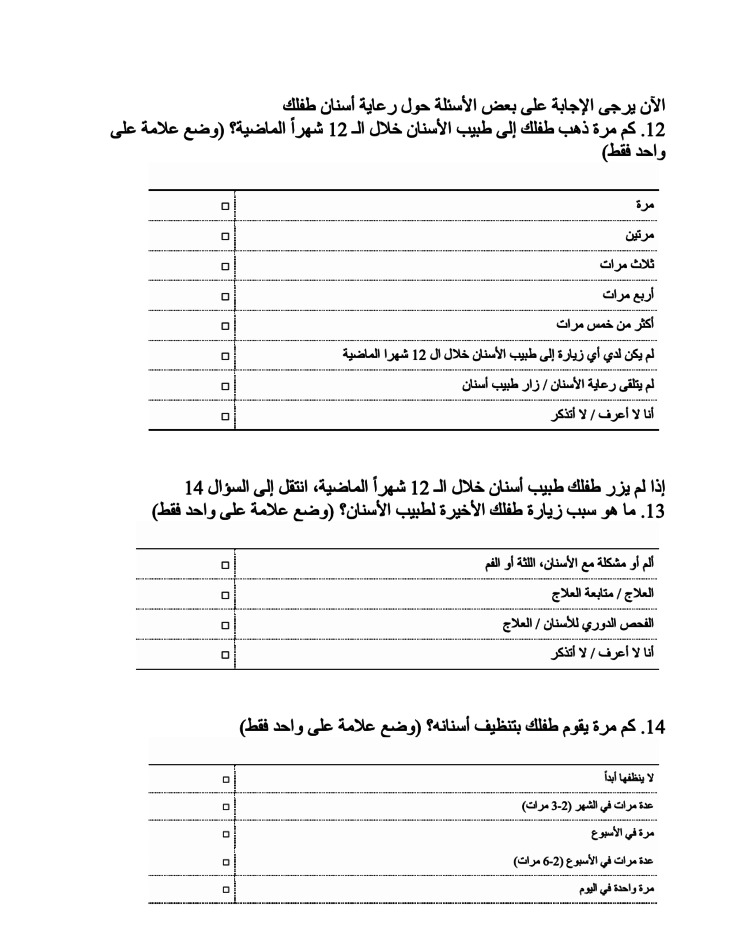
Arabic Questionnaire 4

**Figure 7:**
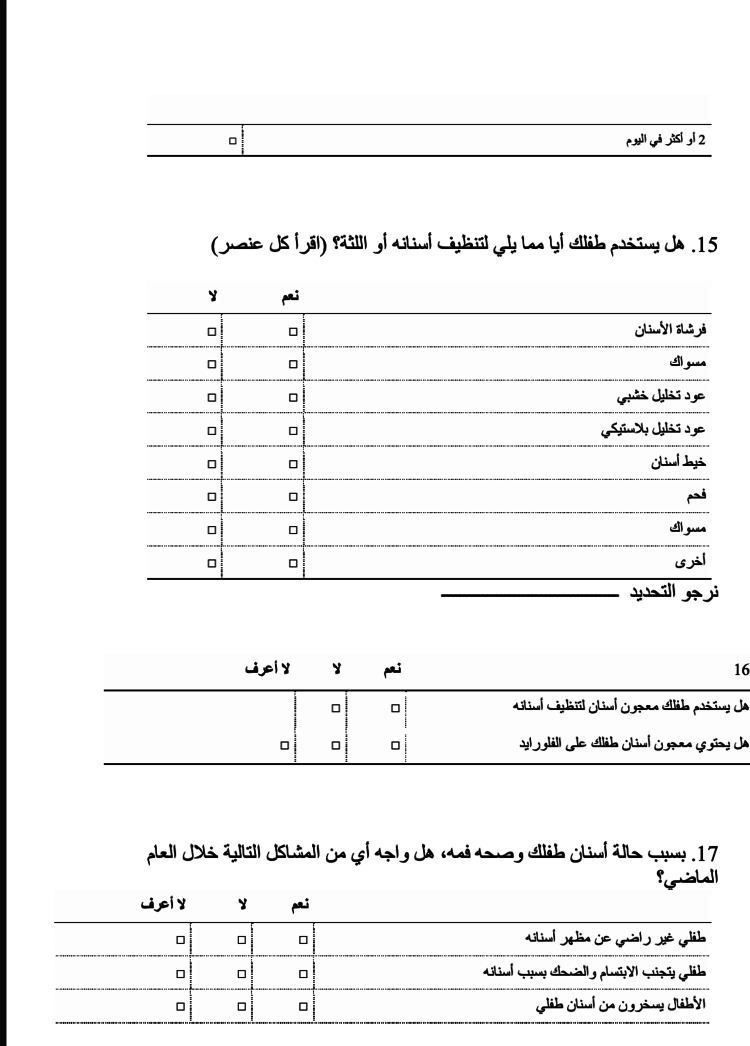
Arabic Questionnaire 5

**Figure 8:**
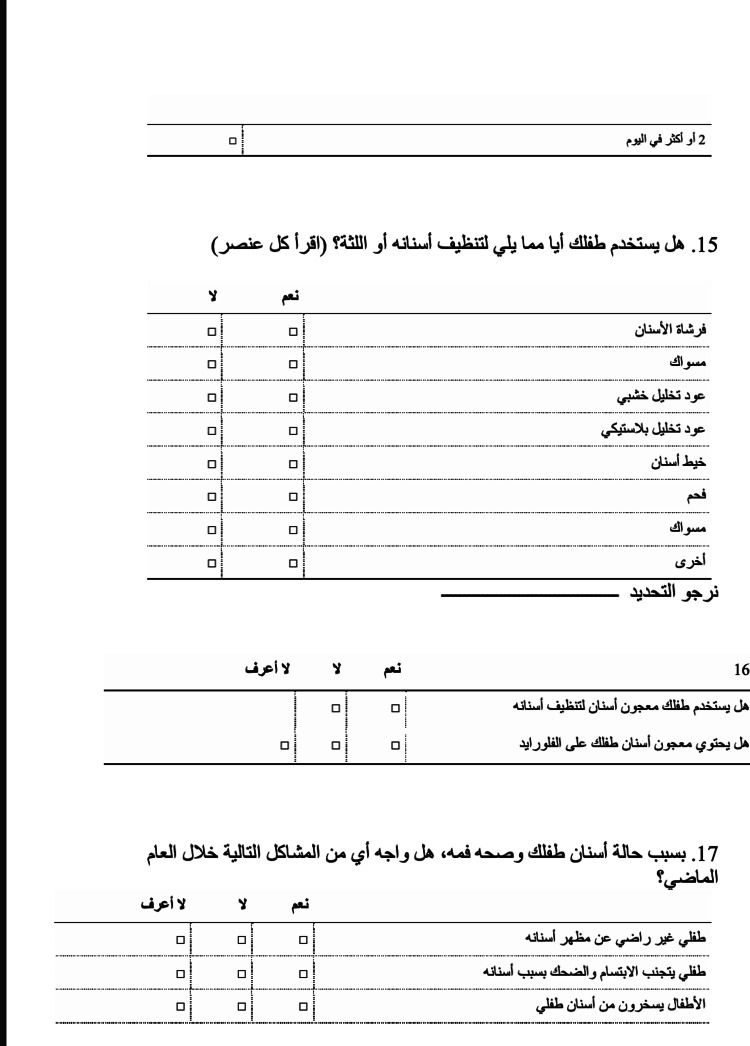
Arabic Questionnaire 6

**Figure 9:**
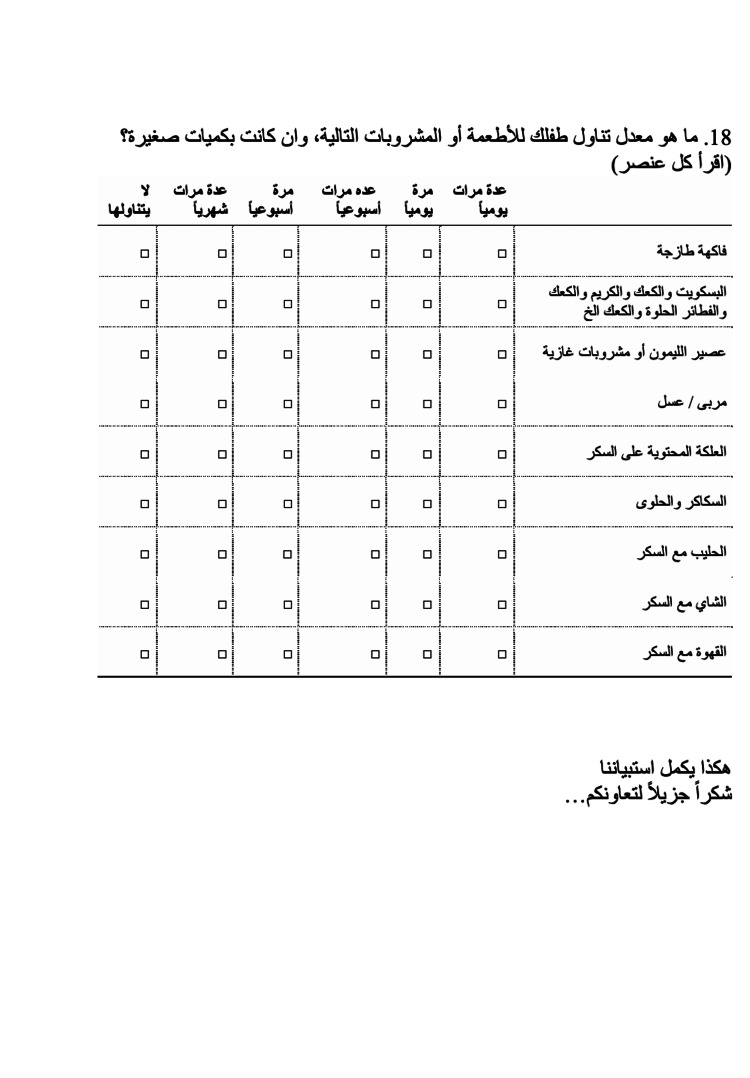
Arabic Questionnaire 7

**Figure 10:**
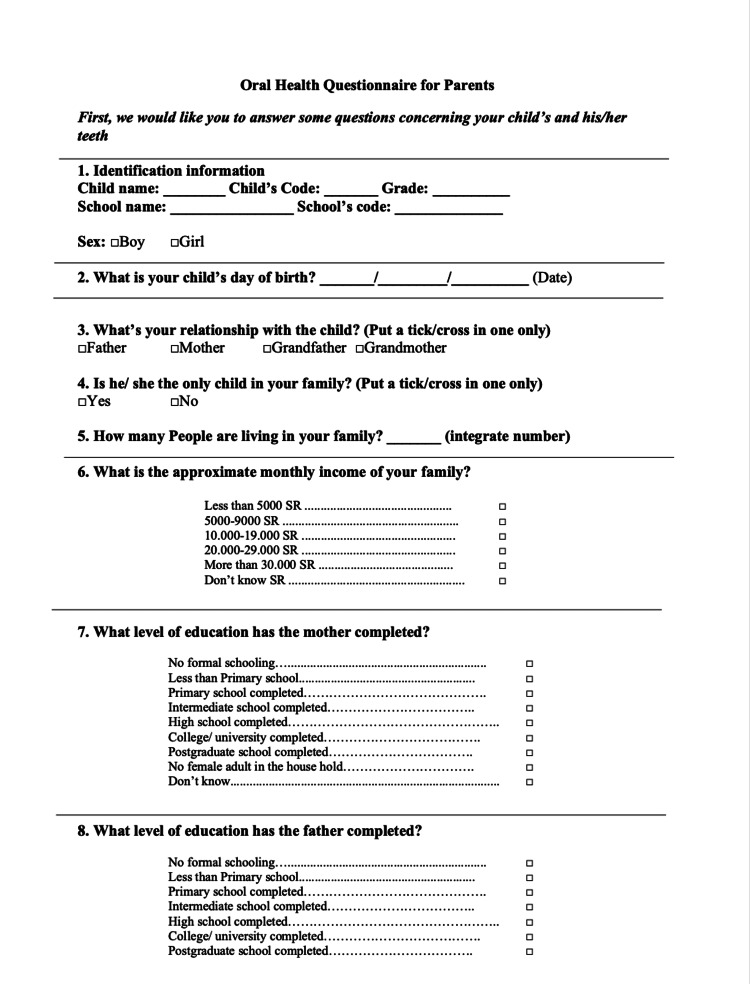
English Questionnaire 1

**Figure 11:**
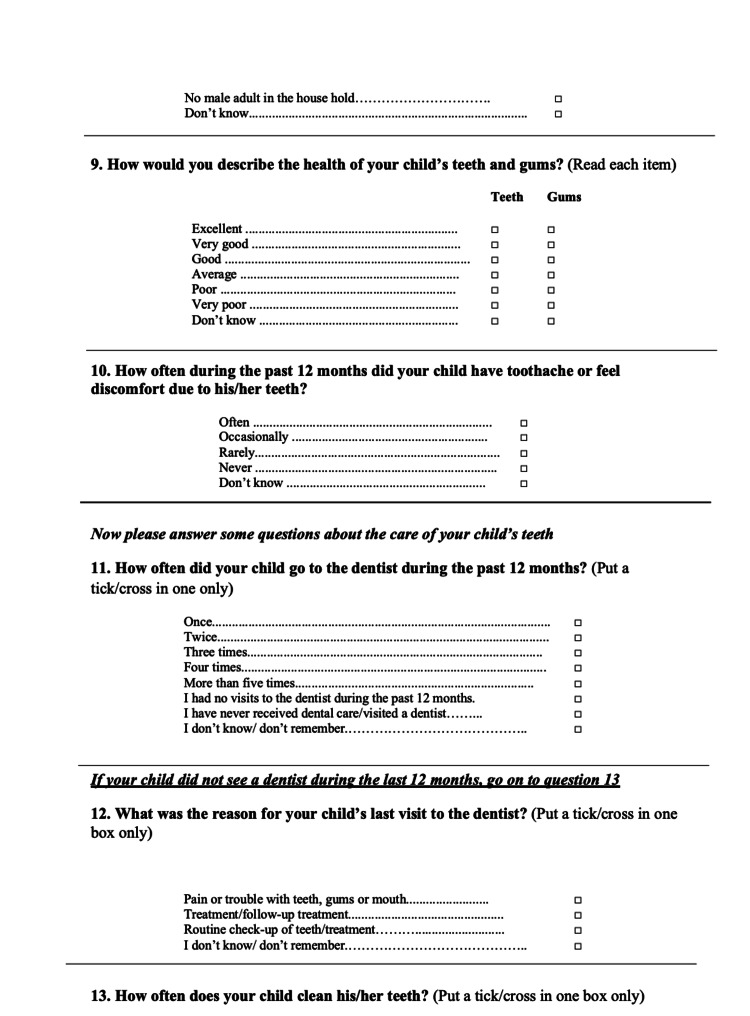
English Questionnaire 2

**Figure 12:**
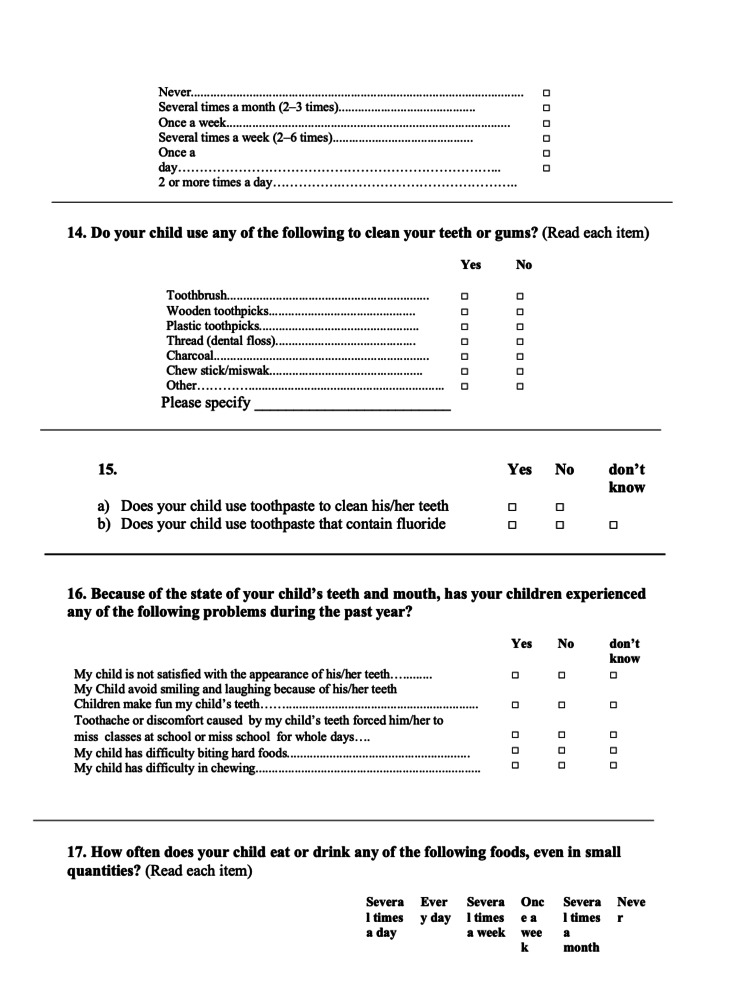
English Questionnaire 3

**Figure 13:**
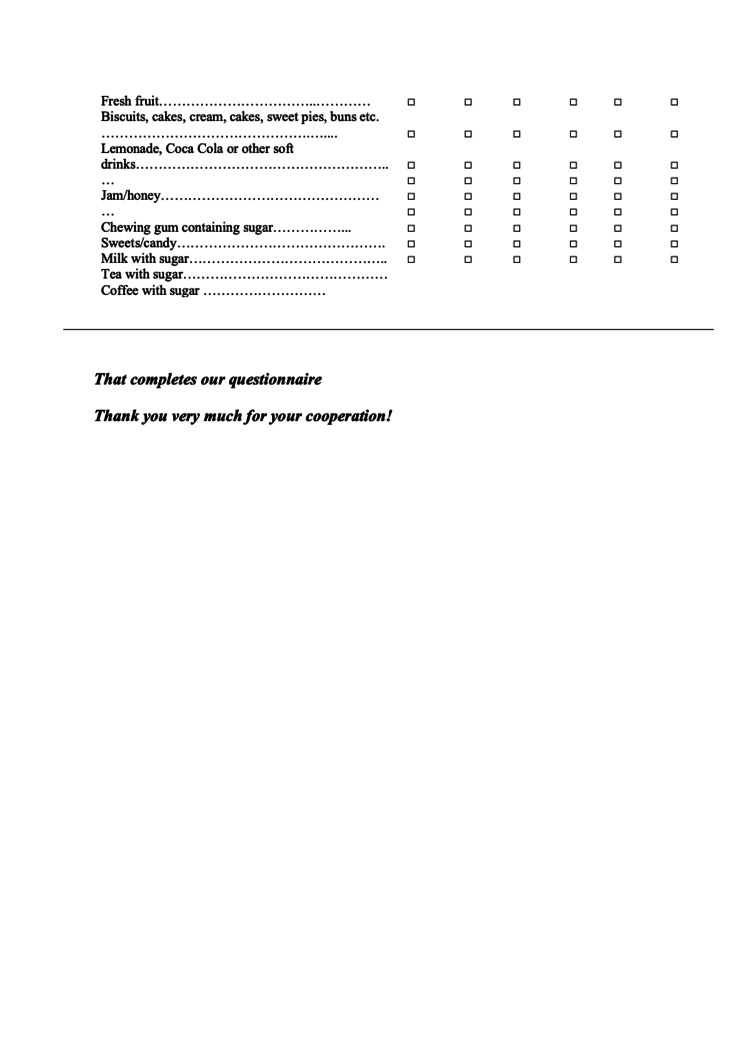
English Questionnaire 4
